# Triple stent-in-stent placement of novel 6-mm multi-hole covered self-expandable metal stents for malignant hilar biliary obstruction

**DOI:** 10.1055/a-2641-2204

**Published:** 2025-07-25

**Authors:** Haruka Toyonaga, Arata Oka, Takuya Takayama, Makoto Masaki, Tatsuya Nakagawa, Hironao Matsumoto, Masaaki Shimatani

**Affiliations:** 1 50196 Department of Gastroenterology and Hepatology, Kansai Medical University Medical Center, Osaka, Japan

Malignant hilar biliary obstruction (MHBO) remains a major challenge in endoscopic management due to its complex anatomy and need for effective segmental drainage. Various approaches have been proposed, including plastic versus metal stents, uncovered versus fully covered self-expandable metal stents (SEMSs), and deployment methods such as stent in stent (SIS) or side by side. However, each strategy has limitations, and no consensus exists regarding the optimal approach 1 .

A covered SEMS with multiple side holes, termed multi-hole covered SEMS (MH-SEMS), was recently developed to address issues such as side branch occlusion, tumor ingrowth, and stent migration, while maintaining removability 2 . Initially designed for distal malignant strictures, its use in hilar lesions has been increasingly reported 3 4 . Until recently, only 10-mm versions were available. The recent introduction of a 6-mm MH-SEMS (HANAROSTENT Benefit Multi-Hole Biliary, M.I.Tech, Korea) allows safe and effective deployment even in narrow and complex intrahepatic ducts ( Fig. 1 , Video 1 ) 5 .

**Fig. 1:**
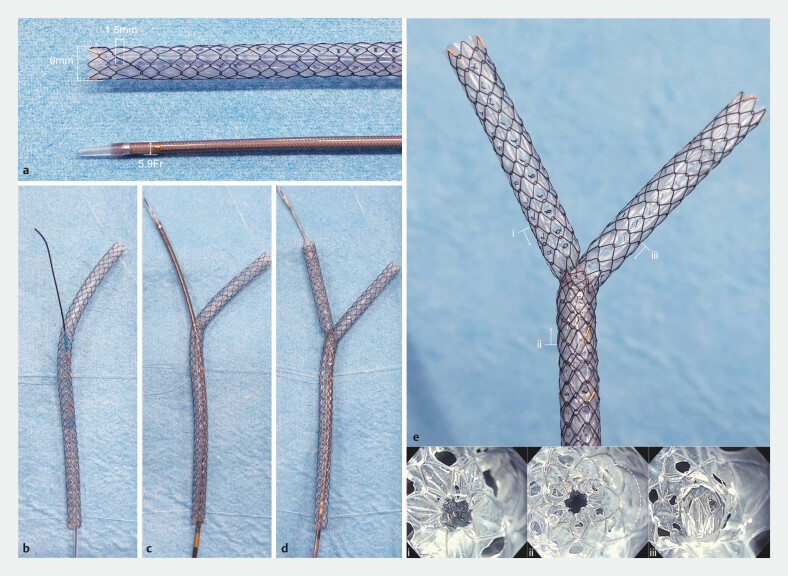
**a** The 6-mm MH-SEMS is equipped with a 5.9-Fr delivery system and features multiple 1.5-mm diameter holes in its covering membrane. This design combines the advantages of both fully covered and uncovered SEMSs, allowing for prevention of side branch obstruction, reduced risk of migration, removability, and resistance to tumor ingrowth. **b–d** In malignant hilar biliary obstruction, the 6-mm diameter and multi-hole design of the stent allows placement not only by the side-by-side technique but also by the stent-in-stent approach. Guidewire access and advancement of a second MH-SEMS delivery sheath through the hole from within the deployed stent can be achieved with ease. **e** MH-SEMSs following stent-in-stent placement. **i** Lumen of the second MH-SEMS. Although the diameter is slightly reduced due to passage through a side hole, the lumen remains smooth and patent. **ii** View of the stent lumen from the distal side. The lumen of the first MH-SEMS is not directly visible due to coverage by the second SEMS, but it is located within the dotted circle. Multiple side holes are identifiable. **iii** Lumen of the first MH-SEMS. The covering membrane of the second MH-SEMS is seen frontally, but bile drainage is maintained through the side hole.

Triple stent-in-stent placement using novel 6-mm multi-hole covered SEMSs enabled effective tri-sectoral drainage in complex malignant hilar biliary obstruction with preserved side branch biliary flow.Video 1

We present a case of a 70-year-old man with unresectable pancreatic cancer and MHBO due to liver metastases ( Fig. 2 ). He developed jaundice caused by occlusion of previously placed plastic stents. Imaging revealed dilation of the left hepatic duct and both the right anterior and posterior sectoral ducts ( Fig. 3 ). Endoscopic retrograde cholangiopancreatography (ERCP) with ENBD was performed, followed by triple SIS placement using 6-mm MH-SEMSs.

**Fig. 2:**
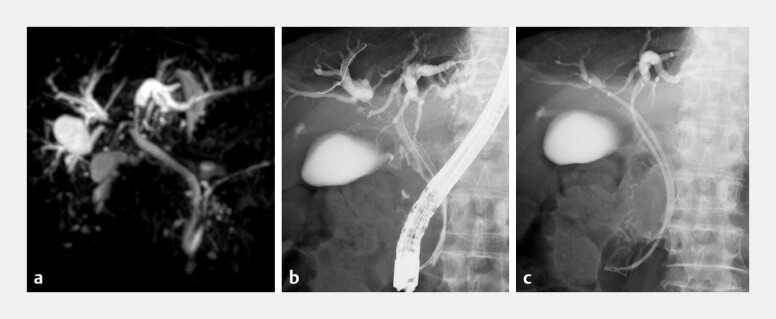
**a** Magnetic resonance cholangiopancreatography (MRCP) showing Bismuth type IIIa malignant hilar biliary obstruction with separate occlusion of the left hepatic duct, right anterior sectoral duct, and right posterior sectoral duct. **b** ERCP confirming the same cholangiographic findings. **c** Bilateral drainage was achieved by placing 7-Fr plastic stents into the left hepatic duct and right anterior sectoral duct.

**Fig. 3:**
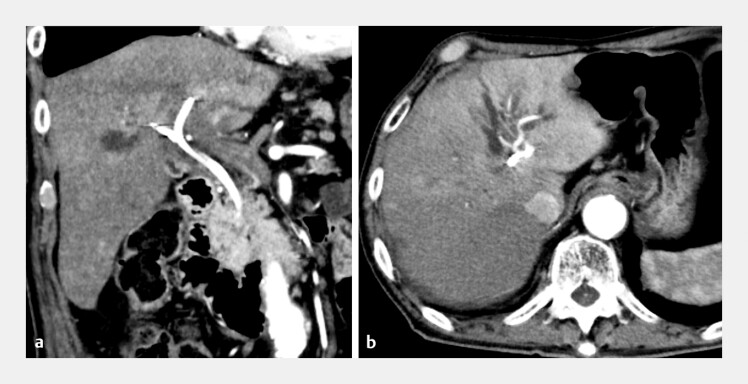
Two months later, the patient developed cholangitis and jaundice. **a, b** Contrast-enhanced CT revealed early enhancement of the left hepatic lobe and dilation of the left intrahepatic bile ducts and both the right anterior and posterior sectoral ducts, indicating stent occlusion.

The first stent was placed in the left hepatic duct, considering the steep angle and infection site. The second was placed into the anterior duct through the side hole, followed by the third into the posterior duct ( Fig. 4 ). This order allowed optional EUS-guided rescue if needed for the posterior duct. Despite the small hole size, guidewire access and delivery were smooth, and bile flow through side branches was preserved ( Fig. 5 ).

**Fig. 4:**
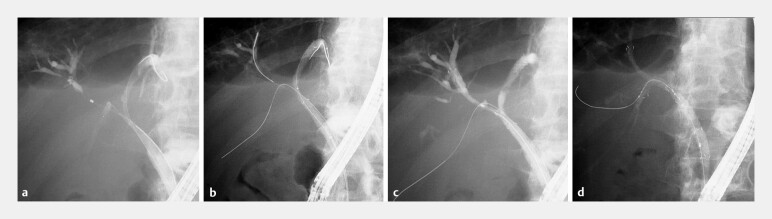
Triple stent-in-stent technique using 6-mm MH-SEMSs for Bismuth type IIIa malignant hilar biliary obstruction. **a** Cholangiography demonstrated the level of obstruction, and guidewires were placed into the left hepatic, right anterior, and right posterior sectoral ducts. **b** The first MH-SEMS was deployed into the left hepatic duct, which had a steep branching angle and was the main site of infection. **c** Although the posterior sectoral duct is typically selected second due to its angulation, it was intentionally reserved for third placement to allow EUS-guided rescue drainage if necessary. The second MH-SEMS was inserted into the anterior sectoral duct through the side hole of the first stent, **d** followed by the third into the posterior sectoral duct, completing triple stent-in-stent placement using MH-SEMSs.

**Fig. 5:**
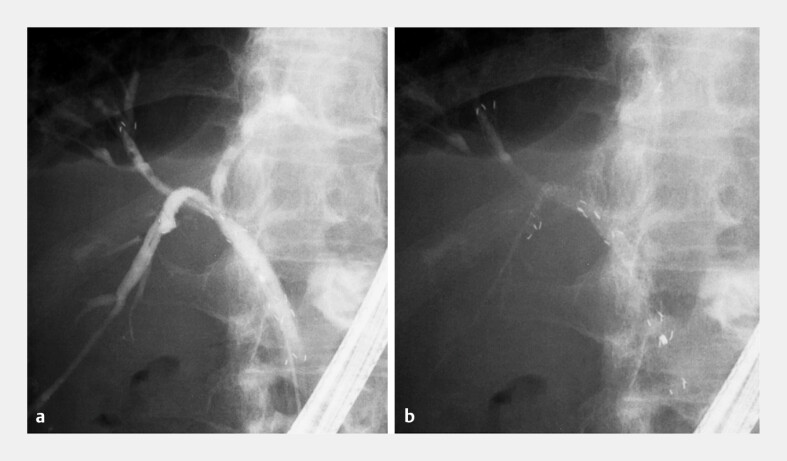
**a** After completion of triple stent-in-stent placement using MH-SEMSs, contrast injection from the distal bile duct demonstrated prompt opacification of all intrahepatic ducts. Additionally, contrast-filled fine biliary branches are located beneath the stent covering through the multi-hole structure. **b** Upon aspiration, contrast was rapidly cleared, confirming effective drainage.

Triple SIS using novel 6-mm MH-SEMSs proved technically feasible and may represent a viable strategy for selective drainage of multiple intrahepatic ducts in complex MHBO.

Endoscopy_UCTN_Code_TTT_1AR_2AZ

## Notes

Endoscopy E-Videos https://eref.thieme.de/e-videos
*E-Videos* is an open access online section of the journal *Endoscopy* , reporting on interesting cases and new techniques in gastroenterological endoscopy. All papers include a high-quality video and are published with a Creative Commons CC-BY license. Endoscopy E-Videos qualify for HINARI discounts and waivers and eligibility is automatically checked during the submission process. We grant 100% waivers to articles whose corresponding authors are based in Group A countries and 50% waivers to those who are based in Group B countries as classified by Research4Life (see: https://www.research4life.org/access/eligibility/ ). This section has its own submission website at https://mc.manuscriptcentral.com/e-videos .

## References

[1] Dumonceau JM Tringali A Papanikolaou IS Endoscopic biliary stenting: indications, choice of stents, and results: European Society of Gastrointestinal Endoscopy (ESGE) Clinical Guideline – Updated October 2017 Endoscopy 2018 50 910 930 10.1055/a-0659-9864 30086596

[2] Takahashi S Takeda T Kobayashi M Efficacy and safety of a novel multi-hole fully covered self-expandable metallic stent for malignant distal biliary obstruction: Multicenter retrospective study Dig Endosc 2025 10.1111/den.15006 40084472

[3] Maruyama H Tanoue K Kurokawa T Stent-in-stent deployment above the papilla to treat malignant hepatic hilar biliary obstruction using novel fully covered multi-hole metal stent Endoscopy 2023 55 E1062 E1064 10.1055/a-2158-7776 37734411 PMC10513778

[4] Ogura T Uba Y Kanadani T Reintervention for recurrent biliary obstruction after stent-in-stent deployment of multi-hole self-expandable metal stents Endoscopy 2025 57 E181 E182 10.1055/a-2534-3143 39978392 PMC11842147

[5] Takahashi S Fujisawa T Takasaki Y Side-by-side placement of a novel slim 6-mm multi-hole covered self-expandable metallic stent for malignant hilar biliary obstruction Endoscopy 2025 57 E312 E313 10.1055/a-2569-7582 40233932 PMC12020676

